# Geometrical Investigation of Piezoelectric Patches for Broadband Energy Harvesting in Non-Deterministic Composite Plates

**DOI:** 10.3390/ma14237370

**Published:** 2021-12-01

**Authors:** Asan G. A. Muthalif, Abdelrahman Ali, Jamil Renno, Azni N. Wahid, Khairul A. M. Nor, Nor Hidayati Diyana Nordin

**Affiliations:** 1 Department of Mechanical and Industrial Engineering, Qatar University, Doha 2713, Qatar; abdelrahman.ali@qu.edu.qa (A.A.); jamil.renno@qu.edu.qa (J.R.); 2 Smart Structures, Systems and Control Research Lab., Department of Mechatronics Engineering, International Islamic University Malaysia, Jalan Gombak, Kuala Lumpur 53100, Malaysia; azni@iium.edu.my (A.N.W.); affendy@iium.edu.my (K.A.M.N.); nhdiyana@iium.edu.my (N.H.D.N.)

**Keywords:** piezoelectric energy harvesters, laminated composites, non-deterministic structures, finite element analysis

## Abstract

Mechanical energy is the most ubiquitous form of energy that can be harvested and converted into useful electrical power. For this reason, the piezoelectric energy harvesters (PEHs), with their inherent electromechanical coupling and high-power density, have been widely incorporated in many applications to generate power from ambient mechanical vibrations. However, one of the main challenges to the wider adoption of PEHs is how to optimize their design for maximum energy harvesting. In this paper, an investigation was conducted on the energy harvesting from seven piezoelectric patch shapes (differing in the number of edges) when attached to a non-deterministic laminated composite (single/double lamina) plate subjected to change in fiber orientation. The performance of the PEHs was examined through a coupled-field finite element (FE) model. The plate was simply supported, and its dynamics were randomized by attaching randomly distributed point masses on the plate surface in addition to applying randomly located time-harmonic point forces. The randomization of point masses and point force location on a thin plate produce non-deterministic response. The design optimization was performed by employing the ensemble-responses of the electrical potential developed across the electrodes of the piezoelectric patches. The results present the optimal fiber orientation and patch shape for maximum energy harvesting in the case of single and double lamina composite plates. The results show that the performance is optimal at 0° or 90° fiber orientation for single-lamina, and at 0°/0° and 0°/90° fiber orientations for double-lamina composites. For frequencies below 25 Hz, patches with a low number of edges exhibited a higher harvesting performance (triangular for single-lamina/quadrilateral for double-lamina). As for the broadband frequencies (above 25 Hz), the performance was optimal for the patches with a higher number of edges (dodecagonal for single-lamina/octagonal for double-lamina).

## 1. Introduction

Ambient mechanical vibration is ubiquitous; harvesting this energy to generate electricity has been considered a pivotal point for researchers in fulfilling the desire for renewable green energy [1,2,3]. Various studies have proposed vibration energy harvesting technologies that can efficiently harness ambient vibration energy and provide sustainable power sources. These technologies demonstrate potential use in powering electronics in a variety of applications, such as micro-powered electronic devices, embedded sensors in structures, medical devices, and wireless sensor networks [4,5,6,7,8]. Moreover, the piezoelectric materials have been used as sensors and/or actuators in the form of layers or patches embedded and/or surface bonded on structural plates for vibration control Bodaghi et al. [9] The energy harvesting mechanism based on piezoelectric energy harvesting has received the utmost interest from many researchers. Piezoelectric energy harvesters (PEHs) are the prominent harvesting systems owing to their high energy conversion capabilities and simple structure [10]. The versatility of PEHs has facilitated their incorporation in various energy harvesting techniques such as fluid-based energy harvesting (Truitt and Mahmoodi, 2013) [11]. Due to the growing interest in PEHs, many investigations have been conducted on the performance optimization of PEHs for optimum power generation. These studies were focused on optimizing the performance of various PEH types, such as the cantilever-type PEH, MEMS-based PEH, and sandwich-type PEH.

Park et al. [12] performed an optimization study on a cantilever-type PEH in which the free tip is excited by the rotary motion of a mechanical device and maximized the power output. Wein et al. [13] performed topology optimization of a cantilever-type PEH using stress norm constraints to optimize the electrical power for a sinusoidal vibrational excitation. Zhu et al. [14] conducted a theoretical and experimental analysis to study the effect of geometrical dimension on the energy harvesting performance of a unimorph cantilever-type PEH with fixed resonance frequency. Kim and Le [15] used a finite element analysis-based topology optimization to calculate the topologies of a substrate plate and a piezoelectric layer in a vibrating cantilever plate application. The designs with optimal topologies were proposed for three different piezoelectric materials, and their voltage output was compared to investigate the most suitable material for the maximum harvesting capacity. Sordo et al. [16] proposed optimizing a MEMS-based PEH by maximizing the number of resonant modes within the narrow operating bandwidth. Song et al. [17] proposed an optimization strategy to maximize the power output density of a PVDF-based cantilever-type energy harvester. This strategy showed that the power output mainly depends on the maximum allowable stress within the beam structure and the device’s working frequency, which can be obtained by adjusting the geometry of the piezoelectric layers. Izadgoshasb et al. [18] explored the effect of the orientation of the cantilever beam structure on the power generation efficiency of the PEH. This type of PEH was proved to have higher voltage output than the conventional PEHs due to the selection of materials and geometry of the core and the metal layers. Kim et al. [19] investigated the performance optimization of cantilever-based PEH by adjusting the position of the piezoelectric generators based on the changes in the phase angle of the substrate. Ichige et al. [20] proposed a PEH with mechanical metamaterials for the elastic layer optimized for high power output and low resonance frequency. Peralta et al. [21] investigated the performance of a bimorph cantilever-type PEH subjected to base excitation to optimize its performance. They performed a parametric study to investigate the effect of shape perturbation on the fundamental frequency, amplitude, and frequency response function.

Most of these studies have investigated the performance optimization of the cantilever-type PEHs at the dominant resonant modes, which are predominantly evident at the low-frequency range. However, investigations on the performance of PEHs at the high-frequency ranges are seldom found in research. Modelling the vibration response of structures at high-frequency ranges is more challenging than the low-frequency range [22]. The vibration response can be divided into three main frequency ranges: low, mid, and high-frequency. The response exhibits dominant resonant peaks at the low-frequency range, and the structure response tends to have a high variance. In contrast, the high-frequency peaks are no longer distinct, and the response variance becomes smaller. The mid-frequency range holds the characteristics of both the low and the high-frequency ranges [23].

**Figure 1:**
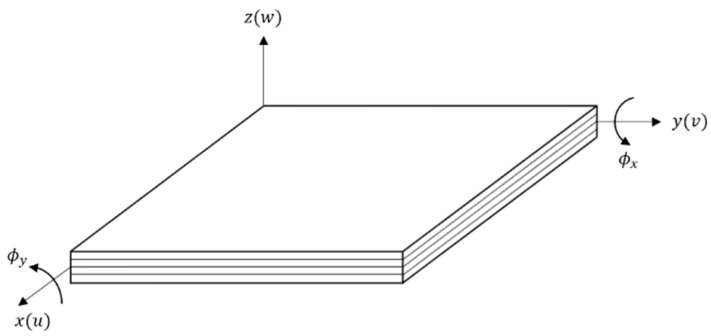
Coordinates of laminated composite plate.

Structures vibrating at the low-frequency range are subjected to long-wavelength deformations and are referred to as deterministic subsystems (DSs); these structures are insensitive to structural uncertainties and can be analyzed using deterministic modelling techniques such as the finite element analysis (FEA). The structures vibrating at high-frequency ranges are subjected to short-wavelength deformations and are sensitive to uncertainties. Such structures are referred to as non-deterministic subsystems (Non-Ds) and analyzed using statistical modelling techniques such as statistical energy analysis (SEA) [24]. The mid-frequency is the range where some components in the system have distinct modal responses and others that behave statistically all in the same frequency range. Therefore, a single modelling technique may not be sufficient to predict the response of such structures [25]. Although the computational resources for FEA can handle smaller elements to capture short wavelengths, it would not be efficient due to computational expenses. Therefore, at high-frequency ranges, SEA is performed to get the ensemble average and predict the response of such vibrations. Hence, SEA is more favourable to use to analyze the dynamics of high-frequency vibrations. In SEA, the modal overlapping factor (MOF) is used to quantify the degree of overlap in the modal response. MOF is defined as the ratio between the modal bandwidth at the half-amplitude and the average modal spacing, and it can be used to establish the three main frequency ranges: low, mid, and high-frequency. For orthotropic plates, the average modal frequency spacing is one of the parameters used in SEA analysis; which can be analytically defined in terms of mechanical properties of the plate using geometric mean of the wave velocities in two principal directions x and y [26]:

(1) $${\bar{\delta f}}_{ortho}=\frac{h}{\sqrt{3}A}\sqrt{c_{Lx}^{’}c_{Ly}^{’}}$$

(2) $$c_{Lx}^{’}=\sqrt{E_{1}/\rho (1-\upsilon_{12}^{2}}),\;c_{Ly}^{’}=\sqrt{E_{2}/\rho \left(1-\upsilon_{21}^{2}\right)}\;$$

where h is the thickness, A is the surface area, while $E_{1}$ and $E_{2}$ are modulus of elasticity, $\upsilon_{12}$ and $\upsilon_{21}$ are Poisson’s ratios in two principal directions, and ρ is the mass density. ($c_{Lx}^{’}$ and $c_{Ly}^{’}$) are the longitudinal wave speed in the x and y directions, respectively (as shown in Figure 1). Equation (1) is only valid for wavelength much greater than the thickness of the plate. The MOF of flexural modes in thin orthotropic plates can be analytically written as [27]:

(3) $$\mathrm{MOF}=\frac{\pi }{2}\frac{f\eta }{\delta f_{ortho}}$$

where η is the damping loss factor and f is the frequency. MOF can be used to indicate the high-frequency threshold value, i.e., MOF=2.5 for plates. The effect of uncertainties due to mass variations, stiffness variations due to boundary conditions, and discontinues has a minimal influence on the low-frequency modes. However, at high-frequency, the structural response is sensitive to such uncertainties. Due to this, the response will be non-deterministic, and the ensemble average is taken. Literature involving energy harvesting have addressed such case; however, the change of the structure anisotropy has not been investigated in the literature.

In this paper, an investigation on the performance of the ceramic-type PEH attached to a non-DS composite plate was conducted using FEA. The investigation focuses on changing the piezoelectric shape and the plate’s anisotropy on the developed voltage. The study proposed seven different piezoelectric patch shapes holding the material properties of the PZT-5A attached to a simply supported laminated composite. Randomly located point masses and time-harmonic point forces were distributed on the host plate to generate uncertainties in the structure, producing the effect of an infinite plate that generates the effect of a non-DS. The investigation was performed by employing the ensemble average response. The electric potential across the piezoelectric patch was obtained, and the ensemble responses were analyzed.

This paper is organized as follows: Section 2 presents the methodology where the mathematical modelling and the FEA model construction are discussed including: composite laminate construction, geometry, material properties, meshing and boundary conditions. Section 3 complies with the ensemble response results obtained from the performed simulations and discusses the response behavior. The paper ends with a summary of the completed work and presents the finding of the research.

## 2. Methodology

The dynamics of the laminated composite plate with the piezoelectric patch attached to it were simulated using ANSYS Mechanical APDL^®^ (ANSYS Inc., Canonsburg, PA, USA). In this section, the mathematical modelling and the FE model construction are presented.

### 2.1. Theory of Laminated Composite Plates

#### 2.1.1. Strain-Displacement Relationship

This section considers a fiber-reinforced laminated composite with distributed piezoelectric patches as sensors bonded on the surface. Figure 1 represents the coordinates of the laminated composite plate. The x-y plane coincides with the mid-plane, whereas the z-axis is normal to the mid-plane.

The displacements field at any point within the laminated composite is given based on the first-order shear deformation plate theory (FSDT) (see Appendix A) by [28]:

(4) $$\begin{array}{c} u\left(x,y,z,t\right)=u_{0}\left(x,y,t\right)+z\phi_{x}\left(x,y,z\right)\\ v\left(x,y,z,t\right)=v_{0}\left(x,y,t\right)+z\phi_{y}\left(x,y,z\right)\\ w\left(x,y,z,t\right)=w_{0}\left(x,y,t\right) \end{array}$$

where u, v and w are the displacements in the x, y, and z directions, and ($u_{0}$, $v_{0}$,$\;w_{0}$) denote the displacements on the plane z=0. While $\phi_{x}$ and $\phi_{y}$ are the rotations of a transverse normal about the y- and x-axes, respectively (they do not follow the right-hand rule):

(5) $$\frac{\partial u}{\partial z}=\phi_{x},\;\;\frac{\partial v}{\partial z}=\phi_{y}$$

In FSDT, the straight lines originally normal to the mid-plane will not remain straight during bending. They will rotate with angular displacements $\phi_{x}$ and $\phi_{y}$ independent of the transverse displacement and its derivatives. This allows the development of stress and shear strains during bending. Additionally, the transverse shear strain is assumed to be constant across the thickness. For thin-plates, the rotation functions $\phi_{x}$ and $\phi_{y}$ approach the respective slopes of the transverse deflections:

(6) $$\phi_{x}=-\frac{\partial w_{0}}{\partial x},\;\;\phi_{y}=-\frac{\partial w_{0}}{\partial y}$$

The nonlinear strains associated with the displacement field in Equation (1) are [29]:

(7) $$\left\{\begin{array}{l} \varepsilon_{xx}\\ \varepsilon_{yy}\\ \gamma_{yz}\\ \gamma_{xz}\\ \gamma_{xy} \end{array}\right\}=\left\{\begin{array}{l} \varepsilon_{xx}^{\left(0\right)}\\ \varepsilon_{yy}^{\left(0\right)}\\ \gamma_{yz}^{\left(0\right)}\\ \gamma_{xz}^{\left(0\right)}\\ \gamma_{xy}^{\left(0\right)} \end{array}\right\}+z\left\{\begin{array}{c} \varepsilon_{xx}^{\left(1\right)}\\ \varepsilon_{yy}^{\left(1\right)}\\ \gamma_{yz}^{\left(1\right)}\\ \gamma_{xz}^{\left(1\right)}\\ \gamma_{xy}^{\left(1\right)} \end{array}\right\}=\left\{\begin{array}{c} \frac{\partial u_{0}}{\partial x}+\frac{1}{2}{\left(\frac{\partial w_{0}}{\partial x}\right)}^{2}\\ \frac{\partial v_{0}}{\partial y}+\frac{1}{2}{\left(\frac{\partial w_{0}}{\partial y}\right)}^{2}\\ \frac{\partial w_{0}}{\partial y}+\phi_{y}\\ \frac{\partial w_{0}}{\partial x}+\phi_{x}\\ \frac{\partial u_{0}}{\partial y}+\frac{\partial v_{0}}{\partial x}\frac{\partial w_{0}}{\partial x}\frac{\partial w_{0}}{\partial y} \end{array}\right\}+z\left\{\begin{array}{c} \frac{\partial \phi_{x}}{\partial x}\\ \frac{\partial \phi_{y}}{\partial y}\\ 0\\ 0\\ \frac{\partial \phi_{x}}{\partial y}+\frac{\partial \phi_{y}}{\partial x} \end{array}\right\}$$

where ($\varepsilon_{xx}^{\left(0\right)}$,$\;\varepsilon_{yy}^{\left(0\right)}$,$\;\gamma_{yz}^{\left(0\right)}$,$\;\gamma_{xz}^{\left(0\right)}$,$\;\gamma_{xy}^{\left(0\right)}$) are the membrane strains, and ($\varepsilon_{xx}^{\left(1\right)}$,$\;\varepsilon_{yy}^{\left(1\right)}$,$\;\gamma_{yz}^{\left(1\right)}$,$\;\gamma_{xz}^{\left(1\right)}$,$\;\gamma_{xy}^{\left(1\right)}$) are the flexural (bending) strains.

#### 2.1.2. Constitutive Equations of a Lamina

In formulating the constitutive equations that represent the mechanical behaviour of a typical lamina of a composite laminate, two assumptions are taken into consideration: (1) no gaps or empty regions are present in the lamina (i.e., lamina is a continuum), and (2) the lamina has a linear elastic behaviour. The first assumption considers the macromechanical/micromechanical behaviour of the lamina, while the latter implies the validation of the generalized Hooke’s law. The laminated plate in Figure 1 has a total thickness h and is composed of a total of N orthotropic layers and the orientation is at angle $\theta_{k}$ to the laminate coordinate x. Certain layers can be for the sensing purpose, i.e., piezoelectric layers. The linear constitutive equations for the kth orthotropic-piezoelectric lamina can be written as [29]:

(8) $$\begin{array}{c} {\left\{\begin{array}{c} \sigma_{1}\\ \sigma_{2}\\ \sigma_{4}\\ \sigma_{5}\\ \sigma_{6} \end{array}\right\}}^{\left(k\right)}={\left[\begin{array}{ccccc} Q_{11} & Q_{12} & 0 & 0 & 0\\ Q_{12} & Q_{22} & 0 & 0 & 0\\ 0 & 0 & Q_{44} & 0 & 0\\ 0 & 0 & 0 & Q_{55} & 0\\ 0 & 0 & 0 & 0 & Q_{66} \end{array}\right]}^{\left(k\right)}\left\{\begin{array}{c} \varepsilon_{1}-\alpha_{1}\Delta T\\ \varepsilon_{2}-\alpha_{2}\Delta T\\ \varepsilon_{4}\\ \varepsilon_{5}\\ \varepsilon_{6} \end{array}\right\}\\ -{\left[\begin{array}{ccc} 0 & 0 & e_{31}\\ 0 & 0 & e_{32}\\ 0 & e_{24} & 0\\ e_{15} & 0 & 0\\ 0 & 0 & 0 \end{array}\right]}^{\left(k\right)}\left\{\begin{array}{c} E_{1}\\ E_{2}\\ E_{3} \end{array}\right\} \end{array}$$

where $Q_{ij}^{\left(k\right)}$ are the elastic stiffnesses, $e_{ij}^{\left(k\right)}$ are the piezoelectric coefficients of the kth lamina, and $\sigma_{i}$, $\varepsilon_{i}$, and $E_{i}$ are the stress, strain, and electric field components, respectively. The thermal expansions are denoted by $\alpha_{1}$ and $\alpha_{2}$, and ΔT is represents the temperature increment from a reference state. It should be noted that for the layers that do not contain piezoelectric materials, the part of the piezoelectric coefficients $e_{ij}^{\left(k\right)}$ in Equation (5) is omitted. The coefficients $Q_{ij}^{\left(k\right)}$ are given in terms of engineering constants of the kth layer as:

(9) $$\begin{array}{c} Q_{11}=\frac{E_{1}}{1-\upsilon_{12}\upsilon_{12}},\;\;Q_{12}=\frac{\upsilon_{12}E_{2}}{1-\upsilon_{12}\upsilon_{12}},\;\;Q_{22}=\frac{E_{2}}{1-\upsilon_{12}\upsilon_{12}},\\ Q_{66}=G_{12},\;\;Q_{22}=G_{23},\;\;Q_{55}=G_{13} \end{array}$$

The laminated composite is made of several orthotropic layers, and their material axes are oriented arbitrarily with respect to the laminate coordinate. Thus, the constitutive equations of each layer must be transformed to the laminate coordinate (x,y,z). Consequently, the stress-strain relations in the laminated coordinates are given as:

(10) $$\left\{\begin{array}{c} \sigma_{xx}\\ \sigma_{yy}\\ \sigma_{yz}\\ \sigma_{xz}\\ \sigma_{xy} \end{array}\right\}=\left[\begin{array}{ccccc} {\bar{Q}}_{11} & {\bar{Q}}_{12} & 0 & 0 & {\bar{Q}}_{16}\\ {\bar{Q}}_{12} & {\bar{Q}}_{22} & 0 & 0 & {\bar{Q}}_{26}\\ 0 & 0 & {\bar{Q}}_{44} & {\bar{Q}}_{45} & 0\\ 0 & 0 & {\bar{Q}}_{45} & {\bar{Q}}_{55} & 0\\ {\bar{Q}}_{16} & {\bar{Q}}_{26} & 0 & 0 & {\bar{Q}}_{66} \end{array}\right]\left(\left\{\begin{array}{c} \varepsilon_{xx}\\ \varepsilon_{yy}\\ \varepsilon_{yz}\\ \varepsilon_{xz}\\ \gamma_{xy} \end{array}\right\}-\left\{\begin{array}{c} {\bar{\alpha }}_{1}\\ {\bar{\alpha }}_{2}\\ 0\\ 0\\ {\bar{\alpha }}_{6} \end{array}\right\}\Delta T\right)-\left[\begin{array}{c} {\bar{e}}_{31}\\ {\bar{e}}_{31}\\ 0\\ 0\\ {\bar{e}}_{36} \end{array}\right]E_{z}$$

where ${\bar{Q}}_{ij}$, (${\bar{\alpha }}_{1}$,$\;{\bar{\alpha }}_{2}$,$\;{\bar{\alpha }}_{3}$), and ${\bar{e}}_{ij}$ are the transformed elastic stiffnesses, thermal coefficients of expansion, and piezoelectric coefficients, respectively:

(11) $$\begin{array}{c} {\bar{Q}}_{11}=Q_{11}\cos^{4}\theta +2\left(Q_{12}+2Q_{66}\right)\sin^{2}\theta \cos^{2}\theta +Q_{22}\sin^{4}\theta \\ {\bar{Q}}_{12}=\left(Q_{11}+Q_{22}-4Q_{66}\right)\sin^{2}\theta \cos^{2}\theta +Q_{12}(\sin^{4}\theta +\cos^{4}\theta )\\ {\bar{Q}}_{22}=Q_{11}\sin^{4}\theta +2\left(Q_{12}+2Q_{66}\right)\sin^{2}\theta \cos^{2}\theta +Q_{22}\cos^{4}\theta \\ {\bar{Q}}_{16}=\left(Q_{11}-Q_{12}-2Q_{66}\right)\sin\theta \cos^{3}+(Q_{12}-Q_{22}+2Q_{66})\;\sin^{3}\theta \;\cos\theta \\ {\bar{Q}}_{16}=\left(Q_{11}-Q_{12}-2Q_{66}\right)\sin\theta \cos\theta +(Q_{12}-Q_{22}+2Q_{66})\;\sin\theta \;\cos^{3}\theta \\ {\bar{Q}}_{66}=\left(Q_{11}+Q_{22}-2Q_{12}-2Q_{66}\right)\sin^{2}\theta \cos^{2}\theta +Q_{66}(\sin^{4}\theta +\cos^{4}\theta )\\ {\bar{Q}}_{44}=Q_{44}\cos^{2}\theta +Q_{55}\sin^{2}\theta \\ {\bar{Q}}_{45}=\left(Q_{55}-Q_{44}\right)\;\cos\theta \;\sin\theta {\bar{Q}}_{55}=Q_{55}\cos^{2}\theta +Q_{44}\sin^{2}\theta \end{array}$$

(12) $$\begin{array}{c} {\bar{\alpha }}_{1}=\alpha_{1}\cos^{2}\theta +\alpha_{2}\sin^{2}\theta \\ {\bar{\alpha }}_{2}=\alpha_{1}\sin^{2}\theta +\alpha_{2}\cos^{2}\theta \\ {\bar{\alpha }}_{1}=2\left(\alpha_{1}-\alpha_{2}\right)\sin\theta \cos\theta \end{array}$$

(13) $$\begin{array}{c} {\bar{e}}_{31}=e_{31}\cos^{2}\theta +e_{32}\sin^{2}\theta \\ {\bar{e}}_{32}=e_{31}\sin^{2}\theta +e_{32}\cos^{2}\theta \\ {\bar{e}}_{36}=\left(e_{31}-e_{32}\right)\sin\theta \cos\theta \end{array}$$

### 2.2. Model Construction

#### 2.2.1. Composite Laminated Plate

The non-DS system proposed in this study consisted of a laminated composite plate with a piezoelectric patch attached to its surface. The laminated composite is an assembly of layers (plies) of fibrous composite materials. The fibers are joined together to provide specific engineering properties, including in-plane stiffness, bending stiffness, and bending strength. The properties of the composite laminate can be significantly altered by changing the orientation of the fibers in each ply. In this study, a rectangular epoxy-glass laminated composite is used as the host plate. Each ply of the epoxy-glass laminate had a thickness of 1 mm. This study proposed two different laminated composite plate models: (i) a single-lamina composite oriented at 0, 45, and 90-degrees. (ii) a double-lamina composite stacked by five different stacking sequences. Table 1 summarizes the fiber orientations of the proposed laminated composite models.

The properties and dimensions of the laminated composite plate in Figure 1 are compiled in Table 2 below.

#### 2.2.2. Piezoelectric Patches

**Figure 2:**
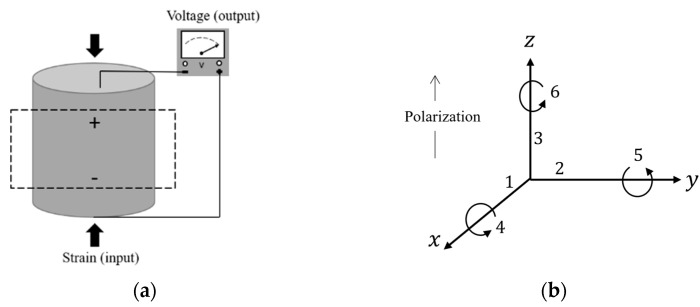
Piezoelectric model. (**a**) Direct piezoelectric effect; (**b**) Directions of forces affecting a piezoelectric element.

PEHs utilize the piezoelectric effect in which an electric charge is induced in response to the applied mechanical strain (i.e., to change in the material polarization). This is referred to as the direct piezoelectric effect. When mechanical stress is applied on the piezoelectric materials, the geometry of their atomic structure changes due to the movement of the positive and negative ions resulting in polarization. Figure 2a presents an illustration of the direct piezoelectric effect.

In linear piezoelectricity, the equations of elasticity are coupled to the charge equation of electrostatics by the mean of piezoelectric electric constants (APDL^®^ adopts IEEE standard form) and the piezoelectric effect can be described in the strain-charge form as:

(14) $$\left\{S\right\}=\left[s^{E}\right]\left\{T\right\}-\left[d^{T}\right]\left\{E\right\}$$

(15) $$\left\{D\right\}=\left[d\right]\left\{T\right\}+\left[\varepsilon^{T}\right]\left\{E\right\}$$

where, {S} is the elastic strain vector, {T} is the stress vector, {E} is the electric field intensity vector and {D} is the electric flux density vector. The matrix $\left[s^{E}\right]$ is the elastic compliance matrix, [d] is the piezoelectric strain matrix and $\left[\varepsilon^{T}\right]$ is the dielectric permittivity matrix at constant stress. For this piezoelectric model, the direction of the positive polarization coincides with the z-axis of the rectangular system (as shown in Figure 2b). The piezoelectric charge constants involved in this model are as follows:

(i) Compression mode $d_{33}$: indicates the polarization generated in the 3-direction per unit of mechanical compression stress applied in the 3-direction to the piezoelectric. Or induced strain in the 3-direction per unit electric field applied in the 3-direction [30,31].
(ii) Transverse Mode $d_{31}$: indicates the polarization in the 3-direction per unit stress applied in the 1-direction. Or, induced strain in the 1-direction per unit electric field applied in the 3-direction [32]
(iii) Shear Mode $d_{15}$: indicates the polarization developed in the 1-direction per unit shear stress 5 applied (shear around the 2-direction) when there are no other external stresses.

**Figure 3:**
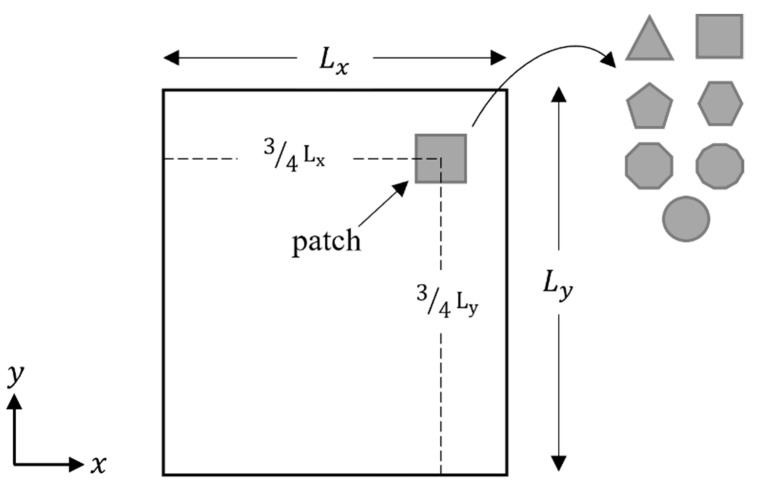
Shapes and location of the piezoelectric patches on the plate.

This study proposed six different piezoelectric patch shapes (varying by the number of edges). The thickness of the piezoelectric patches is 0.5 mm, and the material used in this study is the ceramic-type PZT-5A. The location in which the piezoelectric patches is positioned on the plate was selected to ensure optimum deformation of the piezoelectric patches. It was essential to guarantee that the piezoelectric materials could capture the optimum number of bending modes resulting from the host plate’s deformation. For this reason, the patches were positioned at 3/4 of both the plate’s length and width. Figure 3 shows the shapes and locations of the piezoelectric patches. The material properties and dimensions of the piezoelectric models are compiled in Table 3 below.

#### 2.2.3. FE Model Components and Randomization

**Figure 4:**
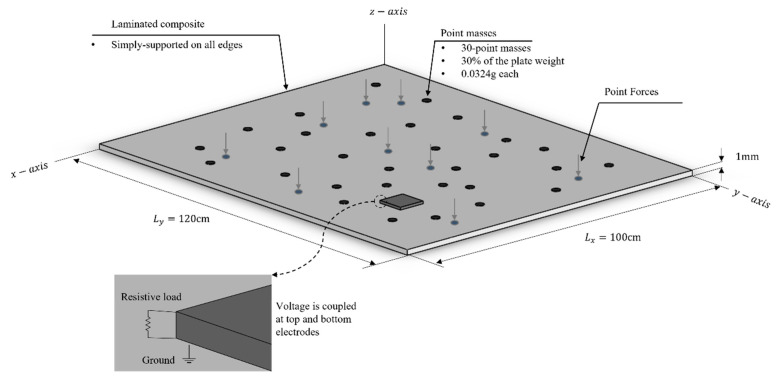
Benchmark model.

The FE model consisted of a simply-supported laminated composite (epoxy-glass) with a ceramic-type piezoelectric patch holding the material properties of PZT-5A. Two different models were proposed throughout this study: a single-lamina and a double-lamina composite. The fiber orientation of the single-lamina composite was varied by $0^{{}^{\circ}}$, $45^{{}^{\circ}}$, and $90^{{}^{\circ}}$. The double-lamina composite had two layers with fiber orientations of: $0^{{}^{\circ}}/0^{{}^{\circ}}$, $0^{{}^{\circ}}/45^{{}^{\circ}}$, $0^{{}^{\circ}}/90^{{}^{\circ}}$, $45^{{}^{\circ}}/90^{{}^{\circ}}$, and $45^{{}^{\circ}}/45^{{}^{\circ}}$. Six different piezoelectric patch shapes were proposed, differing in the number of edges. Figure 4 presents the benchmark model adopted in this study.

#### 2.2.4. Randomization

To promote a dynamic randomization condition to the structure, 30 point masses were randomly attached to the top surface of the plate. The plate is then excited by 20 randomly located time-harmonic point forces, as shown in Figure 4. This randomized condition generated structural uncertainties which in turn produced the effect of an infinite plate. Hence, the real-life response found in non-DS was approached; where the structural response at the high-frequency became sensitive to such uncertainties. Ultimately, the ensemble responses were obtained, and the average response was used for the investigation.

#### 2.2.5. Elements and Mesh

**Figure 5:**
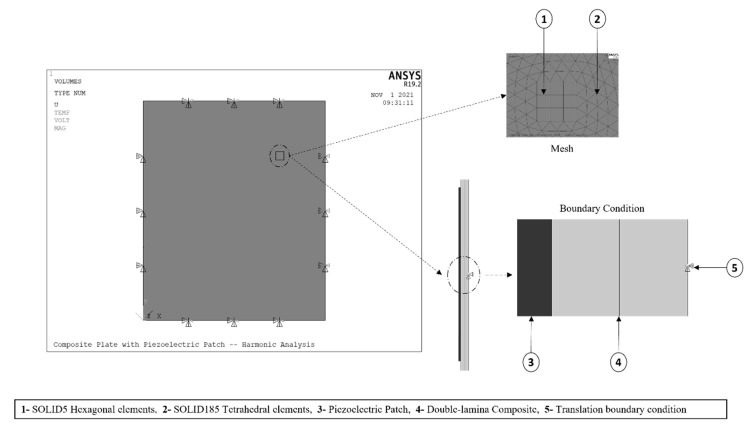
FE Model mesh and boundary condition.

ANSYS was used for the meshing and simulation, as shown in Figure 5. The FE model was meshed using four different element types: SOLID185 was used for the host plate, SOLID5 for the piezoelectric model, MASS21 for the point mass, and CIRCU94 for the resistive load. SOLID185 is used for 3-D modelling of solid structures. It is defined by eight nodes having three degrees of freedom (DOF) at each node (translations in the nodal *x*, *y*, and *z* directions). SOLID5 is a 3-D couple-field solid element and is used for piezoelectric and structural couple-field analysis. This element has eight nodes with up to six DOFs at each node (three translations, one temperature, one voltage, and one magnetic potential). MASS21 is a point element having up to six DOF (three translations and three rotations) and is defined by a single node. As for the CIRCU94 element, it is a circuit element for use in piezoelectric-circuit analyses. This element can interface with SOLID5 to form a harvesting circuit. Information about the selected elements and their capabilities can be found in ANSYS^®^ theory reference [33]

#### 2.2.6. Boundary Condition

The model in Figure 5 has two different boundary conditions: (1) mechanical boundary on the host plate, (2) electrical boundary on the piezoelectric model. The 3-D element used to model the plate has only three translations DOF, and no rotations, the modelling of a simply supported boundary condition can be complicated. Therefore, when considering the plate’s element, the translations DOF of the plate’s lower edges were constrained to approach the effect of a simply-supported boundary condition. As for the electrical boundary condition, the nodes on the upper surface of the piezoelectric patch were coupled to have the same electrical potential throughout the surface; consequently, the surface nodes would act as one. Similarly, the nodes on the bottom surface (electrical ground) were coupled and constrained to zero voltage. Ultimately, the upper electrode of the patch was connected to the bottom electrode (ground) through a very high resistive load. Hence, the electrical potential could be measured at the top piezoelectric surface.

## 3. Results and Analysis

This section presents the results of the ensemble-average responses of the different PEH shapes attached with the non-DS composite laminate. Two different analyses were performed to analyze the response of the harvesting system: (1) modal analysis, and (2) harmonic analysis. The non-DS plate is typically subjected to uncertainties; therefore, one must ensure that a proper frequency range is selected whereby the non-deterministic behaviour can be observed, which is typically noticed at the high-frequency range. A 5% loss factor (η) was selected; this corresponds to a damping ratio (ζ) of 2.5% since η=2ζ. The harmonic analysis was set to a frequency range from 0–800 Hz (800 substeps) with the excitation of the 20 time-harmonic randomly located point forces each of 5N. For the harmonic analysis, 10 runs were performed for each piezoelectric patch shape. The location of the point masses point forces was changed in each run. The 10 responses were then averaged to obtain the ensemble average response. The modal analysis was performed prior to the harmonic analysis to analyze the laminated composite’s natural frequencies and mode shapes. Such analysis was crucial to ensure that the PEH are experiencing the maximum strain at the specified location on the plate; thus, effectively generating maximum power capacity. Additionally, the modal analysis was performed to identify the number of bending modes present within the specified frequency range. Table 4 compiles the natural frequencies and the mode shapes of the composite plate obtained from the modal analysis by the FE method. Full harmonic analysis was performed due to its superior efficiency at high frequencies.

Cumulative frequency analysis was performed to get more insight into the performance of the six patch shapes within different frequency ranges. The cumulative frequency average voltage (CFAV) clearly represents the modes as the value peaks at the region where the resonant modes occur. As the value drops, it means that not many modes are occurring within the respective frequency range. CFAV was obtained by calculating the integral of the voltage response (V) over the studied frequency range (f) as:

(16) $${\mathrm{CFAV}}_{i}=\frac{1}{f_{i}}\overset{f_{i}}{\underset{0}{\int }}V\left(f\right)\;\mathrm{df}\;,{\;\mathrm{where}\;f}_{i}=1,2,3\ldots 800$$

### 3.1. Single-Lamina Composite

#### 3.1.1. Ensemble-Average Voltage Response of Single-Lamina

**Figure 6:**
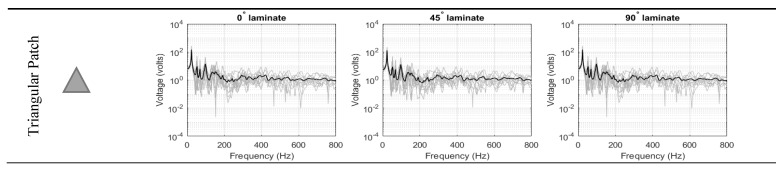

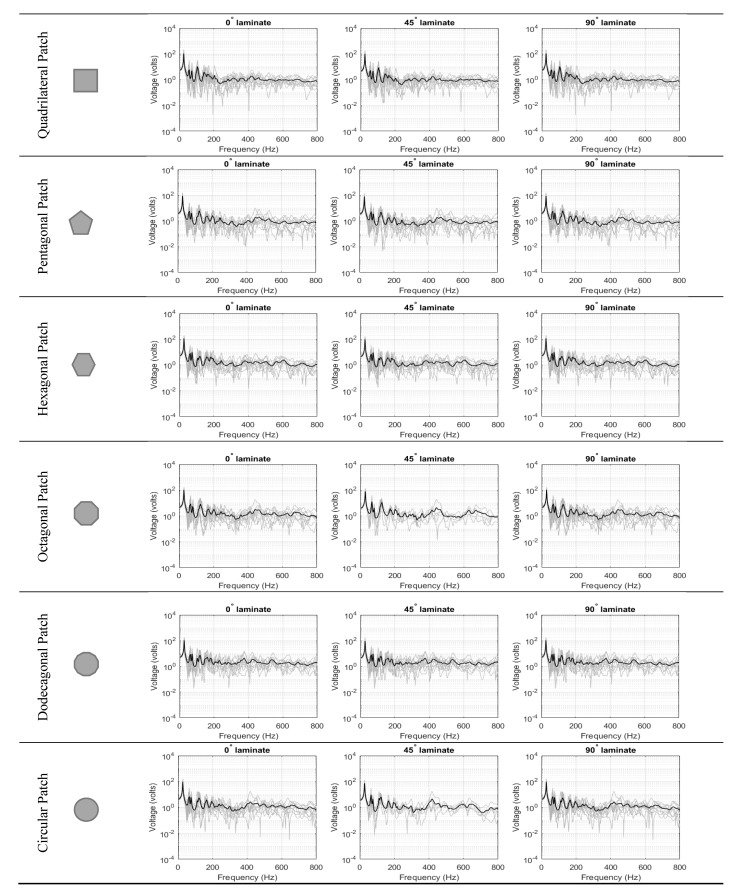
Ensemble-average voltage response for single-lamina.

Figure 6 compiles the ensemble electric potential responses for the 10 randomized structures (for each of the seven piezoelectric patch shapes). The response of each shape was investigated at the three different fiber orientation of the laminated composite: $0^{{}^{\circ}}$,$\;45^{{}^{\circ}}$ and $90^{{}^{\circ}}$. The responses of the PEH hold the characteristics of the typical response of the non-DS structure. The peaks were clearly seen occurring the natural frequencies of the structure. In the low-frequency, the peaks were distinct, well-spaces, and high response variance could be observed. On the other hand, the peaks were no longer distinct at the high-frequency range, and the response demonstrated low response variance. At high-frequencies, the length of the bending wavelength deformation becomes comparable to the distances between the random point masses, and thus, the structure’s response became more sensitive to structural uncertainties, which in turn increases the variance. Compared to previous studies, it is important to note that the piezoelectric voltage did not exceed the breakdown voltage at which the piezoelectric property is lost [34].

**Figure 7:**
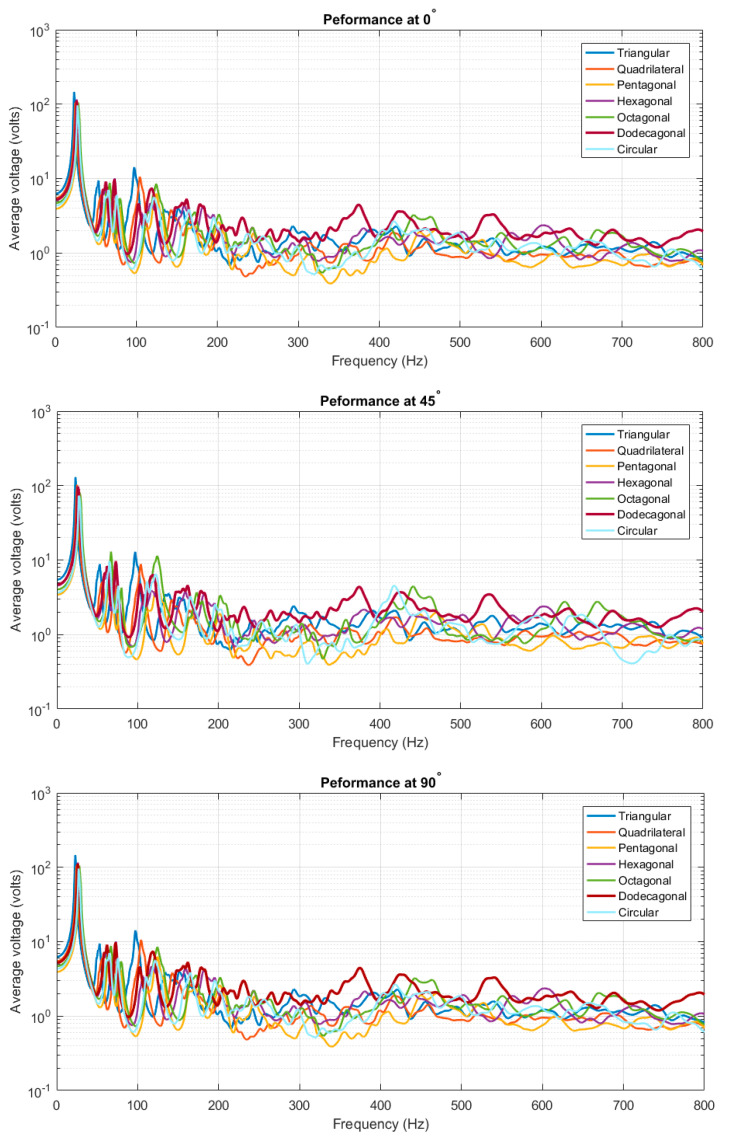
Compiled average response for single-lamina.

The ensemble-average responses are compiled and plotted together for each fiber orientation in Figure 7 below. It could be clearly observed that the ensemble-average response was smoothed out as the system vibrates at the high-frequency, which justifies the purpose of using the SEA method to predict/analyze the response of the non-DS at higher-frequencies.

#### 3.1.2. CFAV of Single-Lamina for Optimal Fiber Orientation

**Figure 8:**
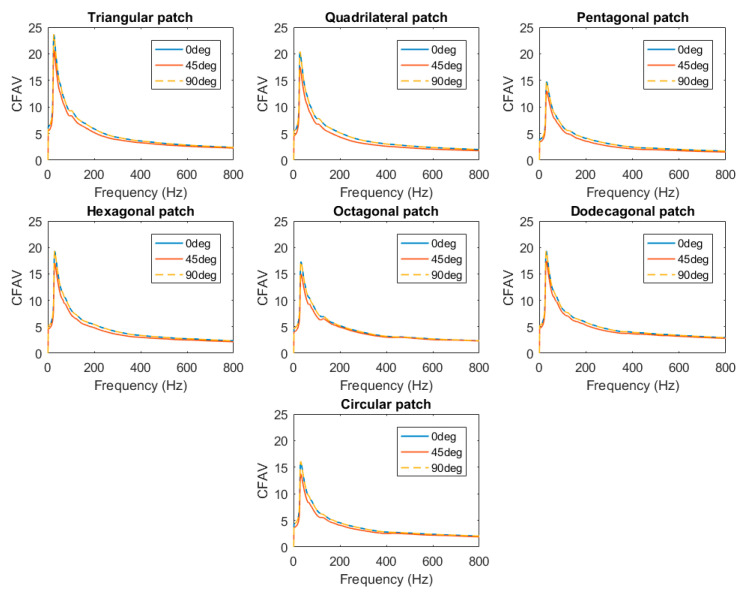
CFAV of the piezoelectric patches for optimal fiber orientation for single-lamina.

This analysis was performed to identify the optimal orientation of fibers used within the laminated composite, as shown in Figure 8. The CFAV was calculated over the entire frequency range for each fiber orientation for each piezoelectric patch shape. It was deduced that the performance differs from one orientation to the other; with the $0^{{}^{\circ}}$ and $90^{{}^{\circ}}\;$fiber orientations having a higher performance than that of the $45^{{}^{\circ}}$. However, it could be clearly noticed that the harvesting performance at both the $0^{{}^{\circ}}$ and $90^{{}^{\circ}}\;$fiber orientations are homogeneous (for every piezoelectric patch shape).

#### 3.1.3. CFAV of Single-Lamina for Optimal Piezoelectric Shape

**Figure 9:**
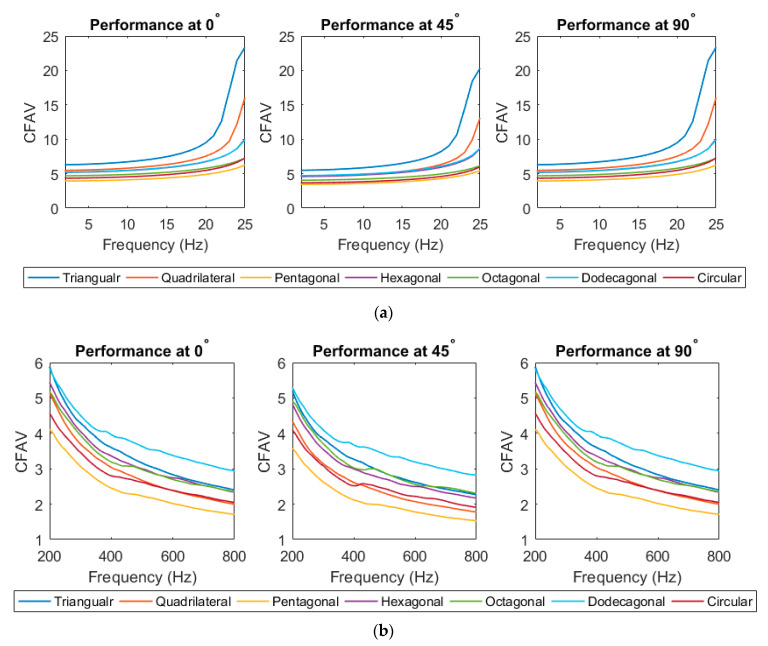
(**a**) frequencies below 25 Hz range (**b**) broadband frequency ranges for single-lamina.

This section calculated the CFAV over the entire frequency range for each of the seven piezoelectric shapes. This analysis was performed to compare the performance of the piezoelectric patch shapes at different fiber orientations. It was deduced that the performance varies at different frequency ranges. Figure 9 below depicts the harvesting performance of the PEHs at different frequency ranges. It was noticed that the piezoelectric patches with a lower number of edges (i.e., triangular, quadrilateral and hexagonal) patches contribute to a higher power generation than those with a higher number of edges. At frequencies below 25 Hz, the triangular patch possessed the highest power generation capacity, followed by the quadrilateral patch. A similar outcome could be realized at different fiber orientations within the low-frequency range. At higher-frequency ranges, it was noticed that the harvesting performance of PEHs with a higher number of edges (i.e., dodecagonal and octagonal) started to increase. The dodecagonal patch contributed to the highest overall CFAV.

### 3.2. Double-Layer Lamina

#### 3.2.1. Ensemble-Average Voltage Response of Double-Lamina

**Figure 10:**
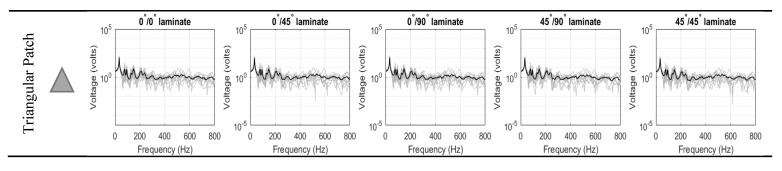

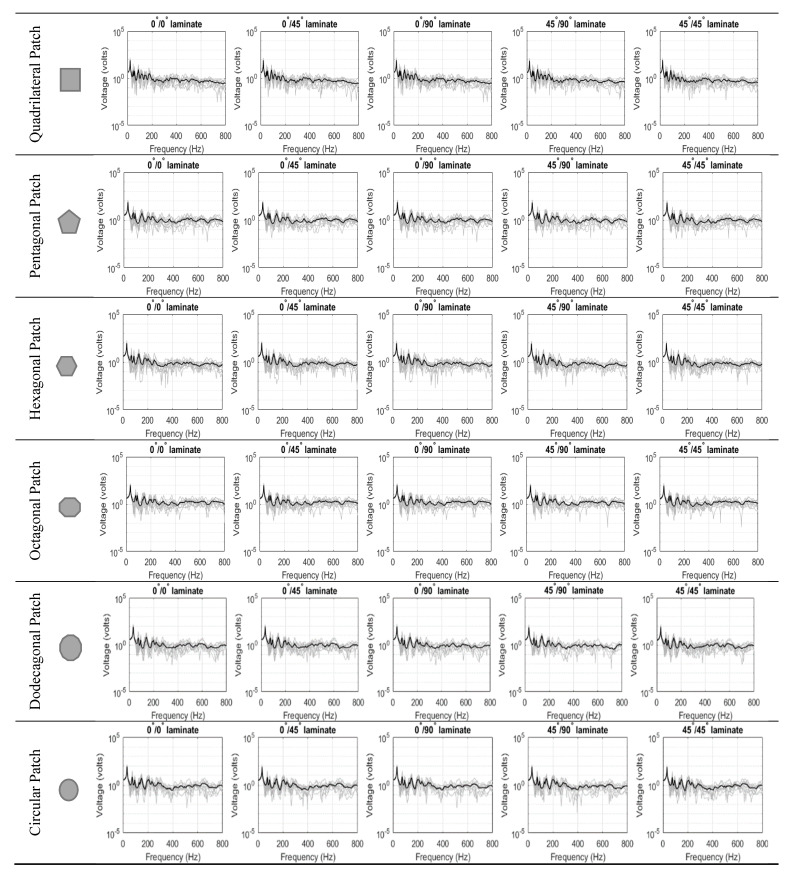
Ensemble-average voltage response for double-lamina.

**Figure 11:**
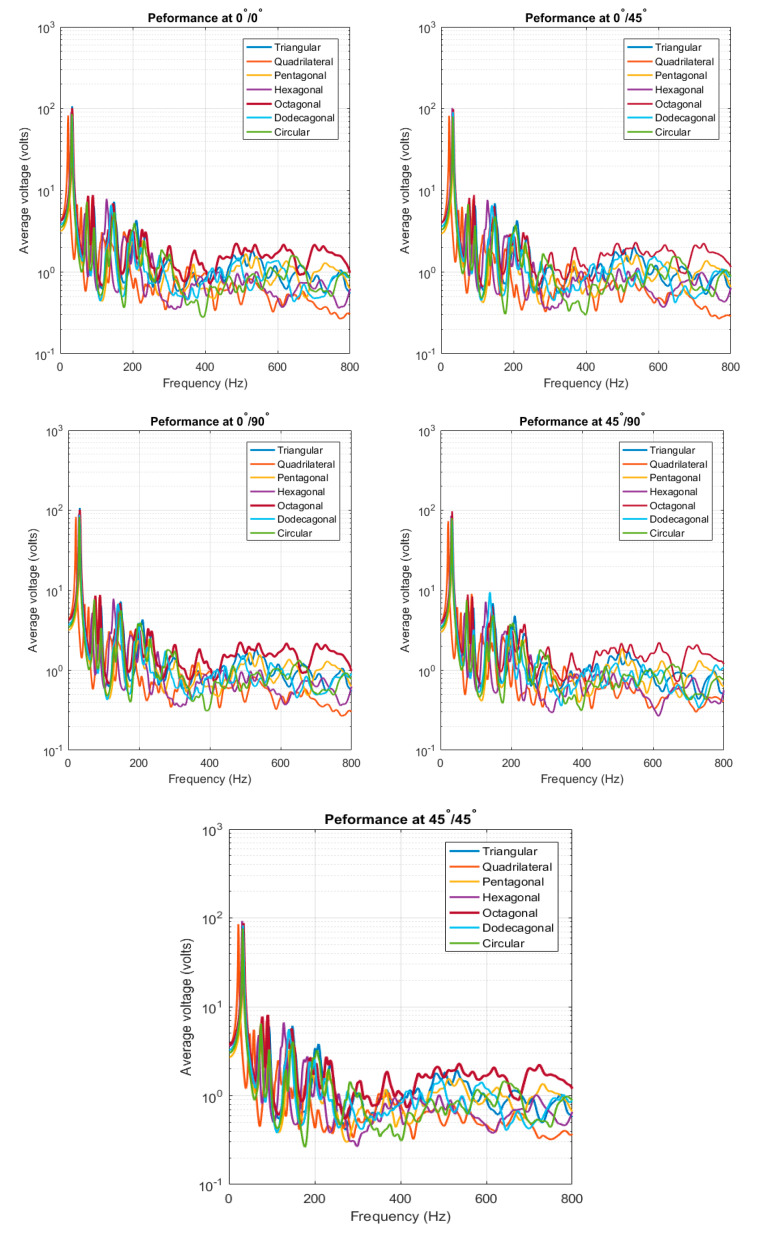
Compiled average-response for double-lamina.

The ensemble-responses of the randomized structures (PEHs attached to the non-DS laminated composite) are presented in Figure 10. The response of each piezoelectric patch shapes was obtained at each stacking sequence with fiber orientation of: $0^{{}^{\circ}}/0^{{}^{\circ}}$,$\;0^{{}^{\circ}}/45^{{}^{\circ}}$,$\;0^{{}^{\circ}}/90^{{}^{\circ}}$,$\;45^{{}^{\circ}}/90^{{}^{\circ}}$, and $45^{{}^{\circ}}/45^{{}^{\circ}}$. The responses exhibited similar characteristics to those found in Figure 6. At the low-frequency, the peaks distinct and contribute to higher amplitudes; whereas, the peaks became broader and indistinct at the high-frequency. In addition, the difference in magnitude between responses at the higher frequency range was not significant. The ensemble-average responses are compiled and plotted together for each fiber orientation in Figure 11 below.

#### 3.2.2. CFAV of Double-Lamina for Optimal FIBER orientation

**Figure 12:**
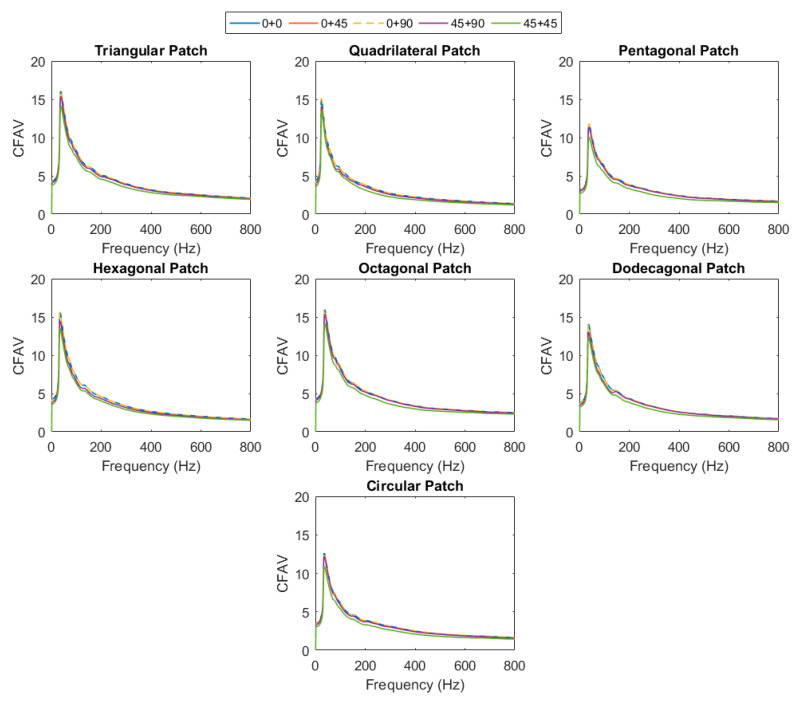
CFAV of the piezoelectric patches for optimal fiber orientation for double-lamina.

In Figure 12, the CFAV was calculated over the entire frequency range to have an insight into the optimal orientation of fibers in the case of double-lamina. It was noticed that the performance of the PEHs when the fibers are orientated at $0^{{}^{\circ}}/0^{{}^{\circ}}$ and $0^{{}^{\circ}}/90^{{}^{\circ}}$ hold the highest harvesting capacities as compared to the other orientations, followed by the fiber orientations of $0^{{}^{\circ}}/45^{{}^{\circ}}$ and $45^{{}^{\circ}}/90^{{}^{\circ}}$. It was found that the performance in the case of the $45^{{}^{\circ}}/45^{{}^{\circ}}$ orientations were the lowest. The performance of the ceramic-type piezoelectric harvesters can be depicted as the patch is operating in the compression $d_{33}$ or the transverse mode $d_{31}$. While the $d_{15}$ is mostly not efficiently used in energy harvesting because its relation is based on shear stress [35]. In the compression mode, the charge constant $d_{33}$ is identified, where the the polarization generated in the 3-direction per unit of mechanical compression stress applied in the 3-direction to the piezoelectric (as shown in Figure 2b). As for the transverse mode, the constant $d_{31}$ indicates the polarization in the 3-direction per unit stress applied in the 1-direction. Both cases are depicted for $0^{{}^{\circ}}$ and $90^{{}^{\circ}}$ fibers orientations where the performance of the PEH is optimal. Hence, it is expected that the performance at $45^{{}^{\circ}}/45^{{}^{\circ}}$ orientation is lower.

#### 3.2.3. CFAV of Double-Lamina for Optimal Piezoelectric Shape

**Figure 13:**
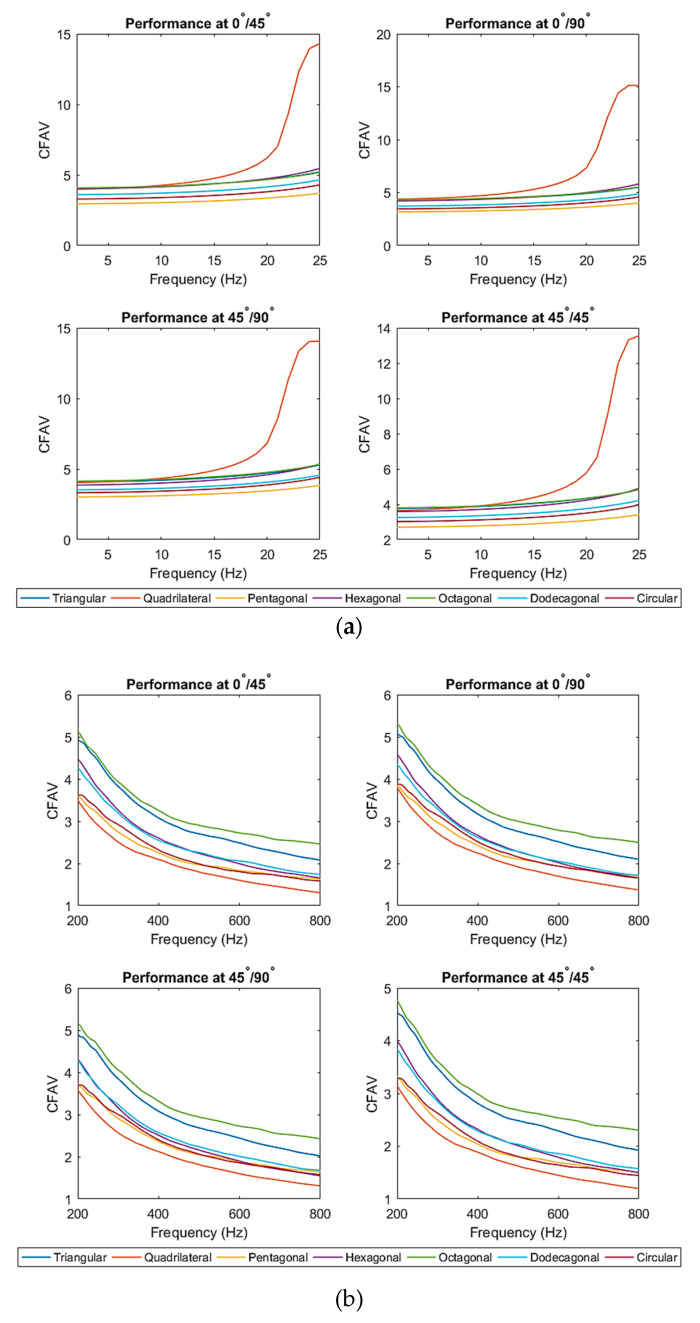
Performance (**a**) frequencies below 25 Hz range (**b**) broadband frequency ranges for double-lamina.

In this section, the total CFAV was calculated for each piezoelectric patch shape and compared at each fiber orientation. It could be noticed that the performance of the PEH is altered when the structure is vibrating in the low and at the high-frequency range. At almost every fiber orientation, it could be deduced the quadrilateral patch possessed the highest harvesting performance as the structure was vibrating at frequencies below 25 Hz, as shown in Figure 13. Patches with a higher number of edges contributed to lower energy harvesting at the low-frequency range. As the structure was vibrating at higher frequency, the performance of the patches with higher number of edges (i.e., octagonal, and dodecagonal) surpassed the quadrilateral patch in the total CFAV. However, an increase in the performance of the triangular patch was detected at the high-frequency range. Overall, it was observed that the octagonal patch contributed to the highest overall CFAV.

**Figure 14:**
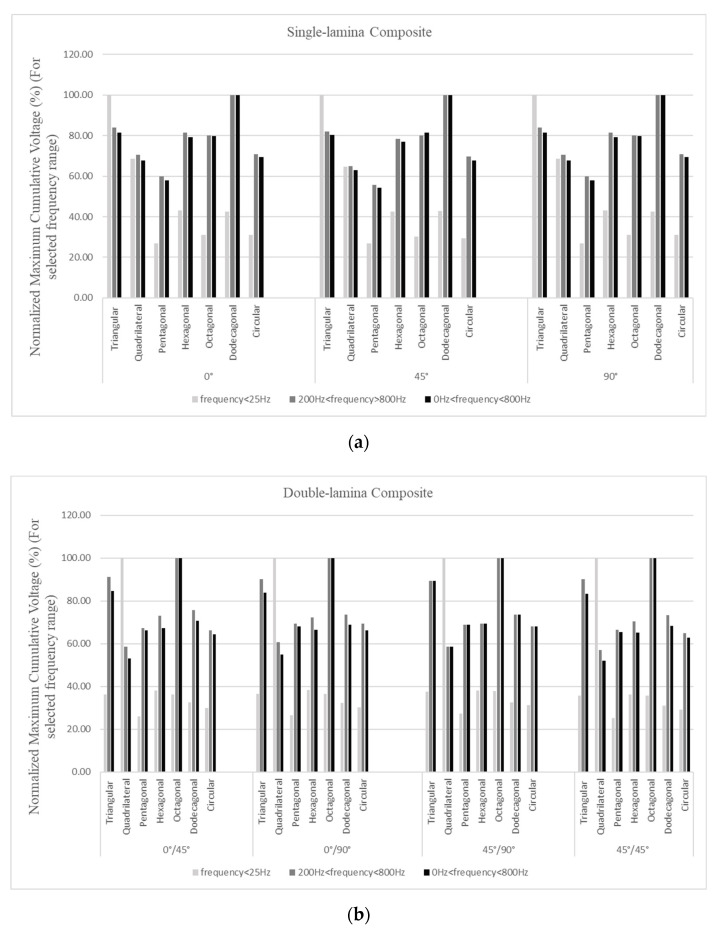
Normalized maximum cumulative voltage (**a**) single-lamina and (**b**) double-lamina composites.

Figure 14 represents the plots for the normalized maximum CFAV for the single and double-lamina composites, where the variation in performance at different frequency ranges can be clearly depicted. For a single layer composite, it is observed that the performance of the triangular patch is higher at lower frequency bands, followed by the quadrilateral patch with almost 30% drop in the performance. The pentagonal patch had the lowest harvesting performance with 75% drop for frequencies below 25 Hz. The dodecagonal patch had the highest performance at intermediate and broadband frequencies, followed by the octagonal patch with around 20% lower harvesting performance.

## 4. Conclusions

In this study, an investigation of the energy harvesting from seven piezoelectric patch shapes (differing in the number of edges) was conducted. PEH models were attached to a non-DS laminated composite plate (single-lamina and double-lamina), and the performance was examined through a coupled-field FE model. The structures’ dynamics were randomized by subjecting the host plate to randomly distributed point masses and randomly located harmonic point forces. The randomized condition generated structural uncertainties required to achieve the effect of an infinite plate structure with the response of a non-DS system. The analysis was performed by employing the ensemble-average responses of the developed electrical voltage across the piezoelectric models. In the case of the single-lamina composite, it was observed that the performance was optimal when the fibers were oriented at either $0^{{}^{\circ}}$ or $90^{{}^{\circ}}$. At frequencies below 25 Hz, the quadrilateral and triangular patches possessed higher harvesting performance. The latter had a higher power generation capacity; however, the patches had a higher number of edges (i.e., dodecagonal, octagonal and hexagonal) exhibited better performance at higher frequency ranges. The dodecagonal patch contributed to the highest overall CFAV in the case of single-lamina composite. As for the double-lamina composite, the performance was found optimal at $0^{{}^{\circ}}/0^{{}^{\circ}}$ and $0^{{}^{\circ}}/90^{{}^{\circ}}$ fiber orientations. The performance of the quadrilateral patch was highest within the low-frequency range but decreased drastically at higher frequency ranges. It was observed that the octagonal patch contributed to the highest overall CFAV for the double-lamina composite. Figure 13 represents the normalized maximum cumulative voltages for single and double-lamina composites.

## Figures and Tables

**Table 1:** Proposed laminated composite models.

Laminated Composite Plate			
Model	Fiber Orientation		
Single-lamina composite	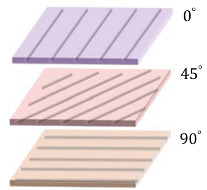		
Double-lamina composite	Model A	$0^{{}^{\circ}}/0^{{}^{\circ}}$	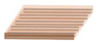
	Model B	$0^{{}^{\circ}}/45^{{}^{\circ}}$	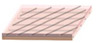
	Model C	$0^{{}^{\circ}}/90^{{}^{\circ}}$	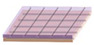
	Model D	$45^{{}^{\circ}}/90^{{}^{\circ}}$	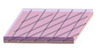
	Model E	$45^{{}^{\circ}}/45^{{}^{\circ}}$	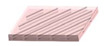

**Table 2:** Material properties and dimensions of the laminated composite.

**Length**		** 100 cm **
**Width**		120 cm
**Ply Thickness**		0.1 cm
**Material**		**Epoxy-Glass**
Modulus of elasticity	$E_{x}$	54 GPa
	$E_{y}$	54 GPa
	$E_{z}$	4.8 GPa
Shear modulus	$G_{xy}$	3.16 GPa
	$G_{yz}$	1.78 GPa
	$G_{xz}$	1.78 GPa
Poisson’s ration	$\upsilon_{xy}$	0.06
	$\upsilon_{yz}$	0.313
	$\upsilon_{xz}$	0.313
Density		2000

**Table 3:** Material properties and dimensions of the piezoelectric models.

Material Property	Coefficient/Shape	Value
Compliance Matrix (**$10^{-12}\;m^{2}$/**N)	$s_{11}=s_{22}$ $s_{12}$ $s_{13}=s_{23}$ $s_{33}$ $s_{44}=s_{55}$ $s_{66}$	16.4 −5.74 −7.22 18.8 47.5 44.3
Relative Permittivity Matrix (**$\varepsilon_{o}=8.854\cdot 10^{-12}\;$**F/m)	$\varepsilon_{11}=\varepsilon_{22}$ $\varepsilon_{33}$	1730 1700
Piezoelectric Strain Matrix (**$10^{-12}$** C/N)	$d_{15}=d_{24}$ $d_{31}=d_{32}$ $d_{33}$	584 −171 374
Density (kg/**m^3^**)	ρ	7750
Side Length (**cm**)	Triangular	6.8
	Quadrilateral	4.5
	Pentagonal	3.4
	Hexagonal	2.8
	Octagonal	2
	Dodecagonal	1.3
	Circular	2.8 radius

**Table 4:** Natural frequencies (Hz) and mode shapes of the composite plate.

	Orientation	Mode 1	Mode 2	Mode 3	Mode 4
**Single**	$0^{{}^{\circ}}$	7.8076	16.281	21.992	26.242
	$45^{{}^{\circ}}$	8.2785	17.486	22.536	29.368
	$90^{{}^{\circ}}$	7.8076	16.281	21.922	26.242
**Double**	$0^{{}^{\circ}}/0^{{}^{\circ}}$	12.265	23.161	30.253	37.537
	$0^{{}^{\circ}}/45^{{}^{\circ}}$	13.764	25.899	31.451	43.243
	$0^{{}^{\circ}}/90^{{}^{\circ}}$	12.265	23.161	30.253	37.537
	$45^{{}^{\circ}}/90^{{}^{\circ}}$	12.019	23.711	30.196	40.927
	$45^{{}^{\circ}}/45^{{}^{\circ}}$	13.263	26.130	31.791	45.171
**Mode Shape**		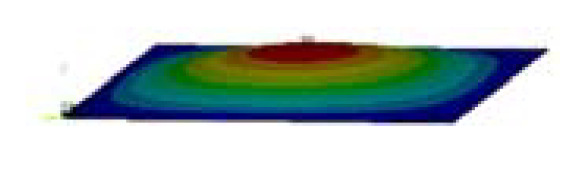	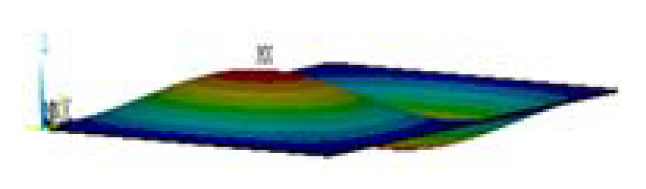	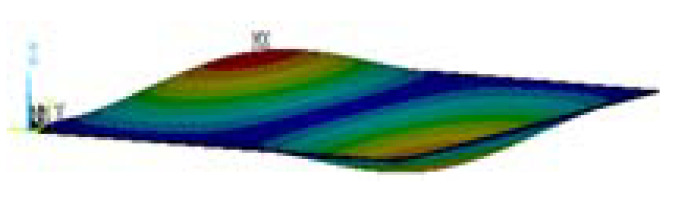	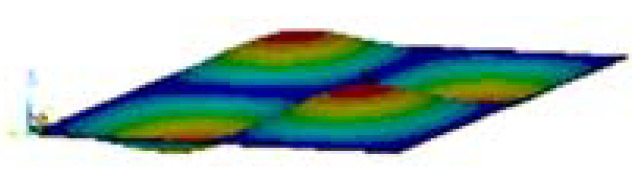
		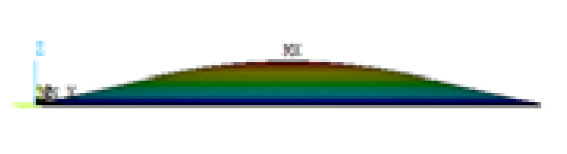	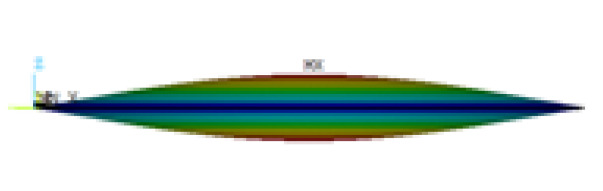	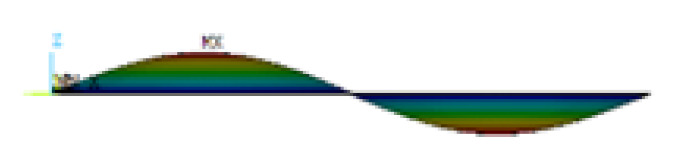	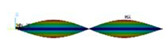

## Data Availability

The data presented in this study are available on reasonable request from the corresponding author.

## Appendix A

The governing equation of motion of the FSDT is obtained using the dynamic version of the principle of virtual displacements:

(A1) $$\frac{\partial N_{xx}}{\partial x}+\frac{\partial Nxy}{\partial y}=I_{0}\frac{\partial^{2}u_{0}}{\partial t^{2}}+I_{1}\frac{\partial^{2}\phi_{x}}{\partial t^{2}}$$

(A2) $$\frac{\partial N_{xy}}{\partial x}+\frac{\partial Nyy}{\partial y}=I_{0}\frac{\partial^{2}v_{0}}{\partial t^{2}}+I_{1}\frac{\partial^{2}\phi_{y}}{\partial t^{2}}$$

(A3) $$\frac{\partial Q_{x}}{\partial x}+\frac{\partial Q_{y}}{\partial y}+N+q=I_{0}\frac{\partial^{2}w_{0}}{\partial t^{2}}$$

(A4) $$\frac{\partial M_{xx}}{\partial x}+\frac{\partial Mxy}{\partial y}-Q_{x}=I_{2}\frac{\partial^{2}\phi_{x}}{\partial t^{2}}+I_{1}\frac{\partial^{2}u_{0}}{\partial t^{2}}$$

(A5) $$\frac{\partial M_{xy}}{\partial x}+\frac{\partial Myy}{\partial y}-Q_{y}=I_{2}\frac{\partial^{2}\phi_{y}}{\partial t^{2}}+I_{1}\frac{\partial^{2}v_{0}}{\partial t^{2}}$$

The quantities ($N_{xx}$,$\;N_{yy}$,$\;N_{xy}$) are the in-plane force resultants, ($M_{xx}$,$M_{yy}$,$\;M_{xy}$) are the moment resultants, and ($I_{0}$,$\;I_{1}$,$\;I_{2}$) are the mass moment of inertia:

(A6) $$\begin{array}{c} \left\{\begin{array}{c} N_{xx}\\ N_{yy}\\ N_{xy} \end{array}\right\}=\int_{-h/2}^{h/2}\left\{\begin{array}{c} \sigma_{xx}\\ \sigma_{yy}\\ \sigma_{xy} \end{array}\right\}dz,\;\left\{\begin{array}{c} M_{xx}\\ M_{yy}\\ M_{xy} \end{array}\right\}=\int_{-h/2}^{h/2}\left\{\begin{array}{c} \sigma_{xx}\\ \sigma_{yy}\\ \sigma_{xy} \end{array}\right\}zdz,\;\left\{\begin{array}{c} I_{0}\\ I_{1}\\ I_{2} \end{array}\right\}=\\ \int_{-h/2}^{h/2}\left\{\begin{array}{c} 1\\ z\\ z^{2} \end{array}\right\}\rho_{0}dz \end{array}$$

(A7) $$=\frac{\partial }{\partial x}\left(N_{xx}\frac{\partial w_{0}}{\partial x}+N_{xy}\frac{\partial w_{0}}{\partial y}\right)+\frac{\partial }{\partial y}\left(N_{xy}\frac{\partial w_{0}}{\partial x}+N_{yy}\frac{\partial w_{0}}{\partial y}\right)$$

The transverse force resultants are denoted by ($Q_{x}$,$\;Q_{y}$) and are given by:

(A8) $$\left\{\begin{array}{c} Q_{x}\\ Q_{y} \end{array}\right\}=K\int_{-h/2}^{h/2}\left\{\begin{array}{c} \sigma_{xz}\\ \sigma_{yz} \end{array}\right\}dz=K\left[\begin{array}{cc} A_{44} & A_{45}\\ A_{45} & A_{55} \end{array}\right]\left\{\begin{array}{c} \begin{array}{c} \gamma_{yz}^{\left(0\right)}\\ \gamma_{xz}^{\left(0\right)} \end{array} \end{array}\right\}$$

where $A_{44}$, $A_{45}$, and $A_{55}$ are the transverse shear stiffnesses and K denotes the shear correction factor; which is used to account for the discrepancy between the actual shear forces and the ones due to the contact stress state predicted by the first-order theory.

The elasticity matrix specifies the stiffness $\left[c_{ij}\right]$ or the compliance coefficients $\left[s_{ij}\right]$. The form used for the elasticity matrix utilizes the compliance form and is input as:

(A9) $$\left[s\right]=\left[\begin{array}{cccccc} s_{11} & s_{12} & s_{13} & 0 & 0 & 0\\ s_{21} & s_{22} & s_{33} & 0 & 0 & 0\\ s_{31} & s_{32} & s_{33} & 0 & 0 & 0\\ 0 & 0 & 0 & s_{44} & 0 & 0\\ 0 & 0 & 0 & 0 & s_{55} & 0\\ 0 & 0 & 0 & 0 & 0 & s_{66} \end{array}\right]$$

The piezoelectric matrix can be input as either a stress matrix [e] or a strain matrix [d]. The stress matrix is associated with the input of the anisotropic elasticity in the stiffness form [c], while the strain matrix corresponds to the input of the elasticity in the compliance form [s]. The piezoelectric matrix is input in the strain from [d] as:

(A10) $$\left[d\right]=\left[\begin{array}{cccccc} 0 & 0 & 0 & 0 & d_{15} & 0\\ 0 & 0 & 0 & d_{24} & 0 & 0\\ d_{31} & d_{32} & d_{33} & 0 & 0 & 0 \end{array}\right]$$

Ansys can convert the piezoelectric strain matrix [d] to a piezoelectric stress matrix [e] using the elasticity matrix [c] by:

(A11) [e]=[c][d]

As for the dielectric matrix, it uses the electric permittivity and is input as a dielectric permittivity matrix at constant stress $\left[\varepsilon^{T}\right]$:

(A12) $$\left[\varepsilon^{T}\right]=\left[\begin{array}{ccc} \varepsilon_{11} & 0 & 0\\ 0 & \varepsilon_{22} & 0\\ 0 & 0 & \varepsilon_{33} \end{array}\right]$$

ANSYS can transform the dielectric matrix at constant stress $\left[\varepsilon^{T}\right]$ to a dielectric matrix at constant strain $\left[\varepsilon^{S}\right]$ by:

(A13) $$\left[\varepsilon^{S}]=[\varepsilon^{T}\right]-{\left[e\right]}^{T}\left[d\right]$$
